# Observation of Skyrmions at Room Temperature in Co_2_FeAl Heusler Alloy Ultrathin Film Heterostructures

**DOI:** 10.1038/s41598-018-35832-3

**Published:** 2019-01-31

**Authors:** Sajid Husain, Naveen Sisodia, Avinash Kumar Chaurasiya, Ankit Kumar, Jitendra Pal Singh, Brajesh S. Yadav, Serkan Akansel, Keun Hwa Chae, Anjan Barman, P. K. Muduli, Peter Svedlindh, Sujeet Chaudhary

**Affiliations:** 1 Thin Film Laboratory, Department of Physics, Indian Institute of Technology Delhi, New Delhi, 110016 India; 2 Department of Condensed Matter Physics and Material Sciences, S. N. Bose National Centre for Basic Sciences, Block – JD, Sector – III, Salt Lake, Kolkata, 700106 India; 3 Department of Engineering Sciences, Uppsala University, SE-75121 Uppsala, Sweden; 4 Solid State Physics Laboratory, Lucknow Road, Timarpur, Delhi, 110054 India; 5 Advanced Analysis Center, Korea Institute of Science and Technology, Seoul, 02792 Republic of Korea

## Abstract

Magnetic skyrmions are topological spin-textures having immense potential for energy efficient spintronic devices. Here, we report the observation of stable skyrmions in unpatterned Ta/Co_2_FeAl(CFA)/MgO thin film heterostructures at room temperature in remnant state employing magnetic force microscopy. It is shown that these skyrmions consisting of ultrathin ferromagnetic CFA Heusler alloy result from strong interfacial Dzyaloshinskii-Moriya interaction (*i*-DMI) as evidenced by Brillouin light scattering measurements, in agreement with the results of micromagnetic simulations. We also emphasize on room temperature observation of multiple skyrmions which can be stabilized for suitable combinations of CFA layer thickness, perpendicular magnetic anisotropy, and *i*-DMI. These results provide a significant step towards designing of room temperature spintronic devices based on skyrmions in full Heusler alloy based thin films.

## Introduction

A novel magnetic pattern in which the magnetic moments exhibit a characteristic swirling configuration is referred to as a skyrmion^1–3^. Remarkably, the skyrmions are topologically stable/protected^4^ structures such that once formed they cannot be deformed/distorted into a ferromagnetic (FM) spin texture or any other magnetic state(s). Due to these properties, skyrmions have immense potential for energy efficient spintronic devices, such as skyrmion based ultra-dense storage, race-track memories, magnetic random access memories (MRAMs), magnetic tunnel junctions, and skyrmion based spin torque oscillators, etc^5–8^. In addition to skyrmions, other magnetically ordered complex spin configurations, *viz*., vortices^9–12^ and magnetic bubbles have also been studied intensively at the nanoscale by the magnetic community^13–15^. Very recently, the skyrmionic bubbles (which possess helicity and vorticity in contrast to chirality in the skyrmions) are proposed in non-centrosymmetric system Fe_3_Sn_2_ which are of large dimensions compared to the skyrmions and have different textures^14,16^. Though skyrmions were first theoretically predicted in the early sixties, their experimental evidence had to wait until 2009 when their presence was reported in non-centrosymmetric B20 compounds like MnSi^1^, FeCoSi^17^, MnGe^18^ and FeGe^19^. However, in them the film thicknesses were several tens of nm and Curie temperatures (T_C_) were lower than 300 K, i.e., 35 K, 29 K, 270 K and 280 K, respectively. On the other hand, the present work is based on the Co_2_FeAl (T_C_ ~1000 K^20^) thin films having perpendicular magnetic anisotropy and skyrmions are observed in a robust manner at room temperature. Further, in all these non-centrosymmetric cases, the samples were cooled to a low temperature and/or required the presence of a high magnetic field to reveal the existence of the skyrmionic state. It was understood that the non-centrosymmetric structures present in these compounds resulted in a non-centrosymmetric spin exchange or the so-called Dzyaloshinskii–Moriya interaction (DMI)^21,22^. From the atomic scale perspective, the DMI energy (E_DM_) is expressed as, ${E}_{DM}=-\,\sum _{i,j}\overrightarrow{{D}_{ij}}\cdot (\overrightarrow{{S}_{i}}\times \overrightarrow{{S}_{j}})$, where $\overrightarrow{{D}_{ij}}$ is the DMI exchange constant characterizing the interaction between the nearest neighbor spins possessing the atomic moments $\overrightarrow{{S}_{i}}$ and $\overrightarrow{{S}_{j}}$. Initially, the DMI was considered to be of bulk origin, however, it was first proposed by Fert *et al*.^23^ that the DMI can be induced in a heavy metal doped system or later by Bogdanov *et al*.^24^ in multilayer structures by breaking the inversion symmetry at the interfaces between FM and nearby non-magnetic ultrathin layers. The so-induced DMI is referred to as interfacial Dzyaloshinskii-Moriya Interaction (*i*-DMI). Since the most striking effect induced by *i*-DMI is the spatial twist of the magnetization leading to the formation of skyrmions even at room temperature^25^, thus, *i*-DMI is expected to be more effective than the bulk DMI in the case of ultrathin films.

Several techniques have been employed to directly confirm the existence of skyrmions. These include sophisticated techniques such as neutron scattering, Lorentz transmission electron microscopy, X-ray holography, spin-polarized STM, etc.^1,26–30^. The studies clearly suggest that the topological nature of the skyrmions makes them very exciting for applications in spintronic devices, primarily due to the giant reduction in the threshold current density by five to six orders of magnitude compared to the current density reported in currently employed FMs for driving the domain walls^31,32^. Even at low current density, the skyrmions are expected to move with high speed especially in materials with low Gilbert damping^33^ such as Heusler alloys which are established for low Gilbert damping^34^. Fert’s group has theoretically predicted the formation of skyrmions by means of micromagnetic simulations in confined media^3^. This non-trivial magnetic spin structure so formed is commonly defined by the skyrmion number, ${N}_{sk}=\frac{1}{4\pi }\int {\bf{n}}.\frac{\delta {\bf{n}}}{\delta x}\times \frac{\delta {\bf{n}}}{\delta y}dxdy$, where **n** is the magnetization unit vector. Based on the magnetization *evolution* structure, the value of *N*_*sk*_ is 1 for the existence of skyrmions, *e*.*g*., spin spiral^1^ or hedgehog type^35^.

Recently, various groups have focused on the ultrathin magnetic film/non-magnetic heavy metal interface^36,37^ to explore the formation of skyrmions. In these studies, there exists a plenty of room for varying the various control parameters, *viz*., material, thickness and the structure of the interfacial layer employed for the control of *i*-DMI, exchange and anisotropy constants, *etc*. By appropriate tuning of the control parameters, the strength of *i*-DMI can be tuned significantly^37^. Rohart *et al*. theoretically predicted the confinement of skyrmions in Pt/Co/AlO_x_ ultrathin films nanostructures exhibiting *i*-DMI^38^. In recent reports, room temperature magnetic skyrmions in Co/Pd^26^, Pt/Co/MgO^25^ and Co/Ni^39^ ultrathin magnetic multilayer structures were reported. For spintronic applications, it is inevitably important to stabilize the skyrmions at room temperature and in absence of external magnetic field. Few reports are also available on the room temperature and zero magnetic field skyrmion^40–42^. To the best of our knowledge, the existence of skyrmions in high spin-polarized materials, *i*.*e*., full Heusler alloys such as Co_2_FeAl (CFA) which possess low damping constant^34^, high Curie temperature^20^, large spin polarization^43^ and tunable spin-dynamics properties^44,45^ is yet to be reported. Despite the rapidly increasing number of experimental studies for gaining further insight into understanding the stabilization of skyrmions in ultrathin films, the robust formation of skyrmions at room temperature in large continuous thin films continues to be an exciting area.

In this report, the direct imaging of stable skyrmions at room temperature employing magnetic force microscopy (MFM) in trilayer stacks containing CFA full Heusler alloy ultrathin films is presented. There exits a few reports on the observation of skyrmions using MFM technique at low temperature as well as few at room temperature^39,46–49^. In the present study, a Heusler alloy ultrathin film based heterostructure Ta/CFA(*t*)/MgO has been used in truly unpatterned form (see methods for growth and measurement details). The motivation for this work lies in the prediction that *i*-DMI originates at the interface and is inversely proportional to the FM layer thickness^37^. The micromagnetic magnetization and MFM simulations performed in the present study also corroborate the existence of skyrmions in these trilayer ultrathin film heterostructures. The simulations clearly demonstrate that the realization of skyrmionic state is indeed conceivable as a result of the competition between the *i*-DMI, anisotropy, exchange interaction and dipolar interaction in Ta/CFA/MgO heterostructure.

## Results and Discussion

**Figure 1:**
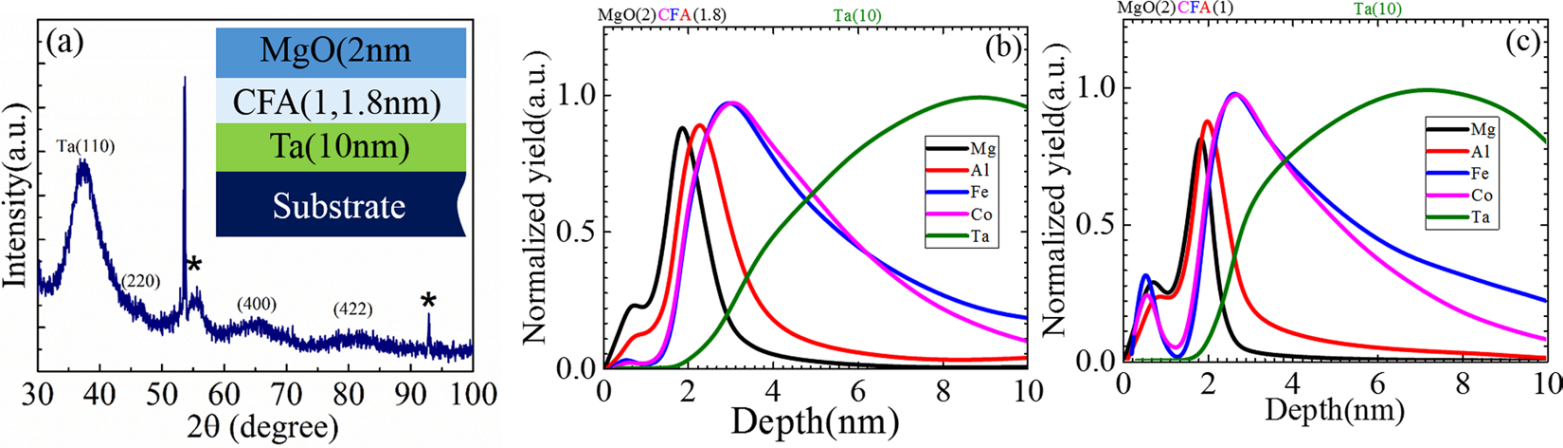
Slow scan GAXRD pattern of (**a**) Si(100)/Ta(10 nm)/CFA(1.8 nm)/MgO(2 nm) heterostructure (peaks marked with * correspond to the Si substrate). Normalized SIMS sputter depth profiles recorded on (**b**) Si/Ta(10)/CFA(1.8)/MgO(2) and (**c**) Si/Ta(10)/CFA(1.0)/MgO(2) hetero-structures. Each color corresponds to different element line profile as indicated in the panel. The profiles of the constituent elements of CFA are similar in both the samples.

Figure 1(a) shows the XRD pattern recorded on Ta(10)/CFA(1.8)/MgO(2) trilayer ultrathin sample. Thickness and elemental analysis of the each layer in the stack have been confirmed by X-ray reflectively (XRR) and X-ray photoelectron spectroscopy (XPS) measurements, respectively (see Supplementary Information S2 and S3). In XRD pattern, it can be seen that while diffraction from Ta(110) is clearly observed, two broad humps appear at diffraction angle of 65.5° and 82.5°, whose position match with the (400) and (422) superlattice reflections, respectively of the CFA [See Fig. 1(a) in ref.^34^].

In order to confirm the chemical/elemental composition in Ta/CFA/MgO trilayer ultrathin films, the secondary ion mass spectroscopy (SIMS) profiles are recorded on the two samples with different CFA thicknesses. Figure 1(b,c) show the depth profiles (normalized intensity) of each element as a function of sample thickness. Profiling was done through the film from the top to down (vertically). As expected, the 2 nm thin MgO can be seen to be the topmost layer (c.f. the left most Mg peak). This is followed by the peaks due to constituent elements Co, Fe and Al of the next 1.8 nm [Fig. 1(b)] and 1.0 nm [Fig. 1(c)] Co_2_FeAl ultrathin films in trilayer heterostructures. Finally, a broad peak due to thicker Ta(10 nm) layer is clearly seen in both the profiles. In the absence of suitable standard reference sample, the absolute atomic concentrations could not be determined. However, a comparison of the intensity of the measured peaks shows that the signals for each element are present in the CFA ultrathin films. The appearance of small shoulder/peak just below 1 nm depth of the sample is quite common and is attributed to the ‘sputtering transition’ associated with the different sputtering yields of elements present in the sample under investigation. It is clear that Co and Fe are uniformly distributed over the film thickness. A small shift of Al-peak could be due to its well-known dominant surface diffusion on account of intermixing due to atomic displacement and minor diffusions that occur within the collision cascade region created along the track of high energy (~2 keV) Cs^+^ ion used for sputter depth profiling. Such interfacial mixing is known to result in the artificial broadening of the profile^50^ as is indeed observed in the recorded depth profiles. From the foregoing, it is thus concluded that in these heterostructures, the CFA exists as an alloy albeit with the possibility of slightly different composition owing to such small film thicknesses.

**Figure 2:**
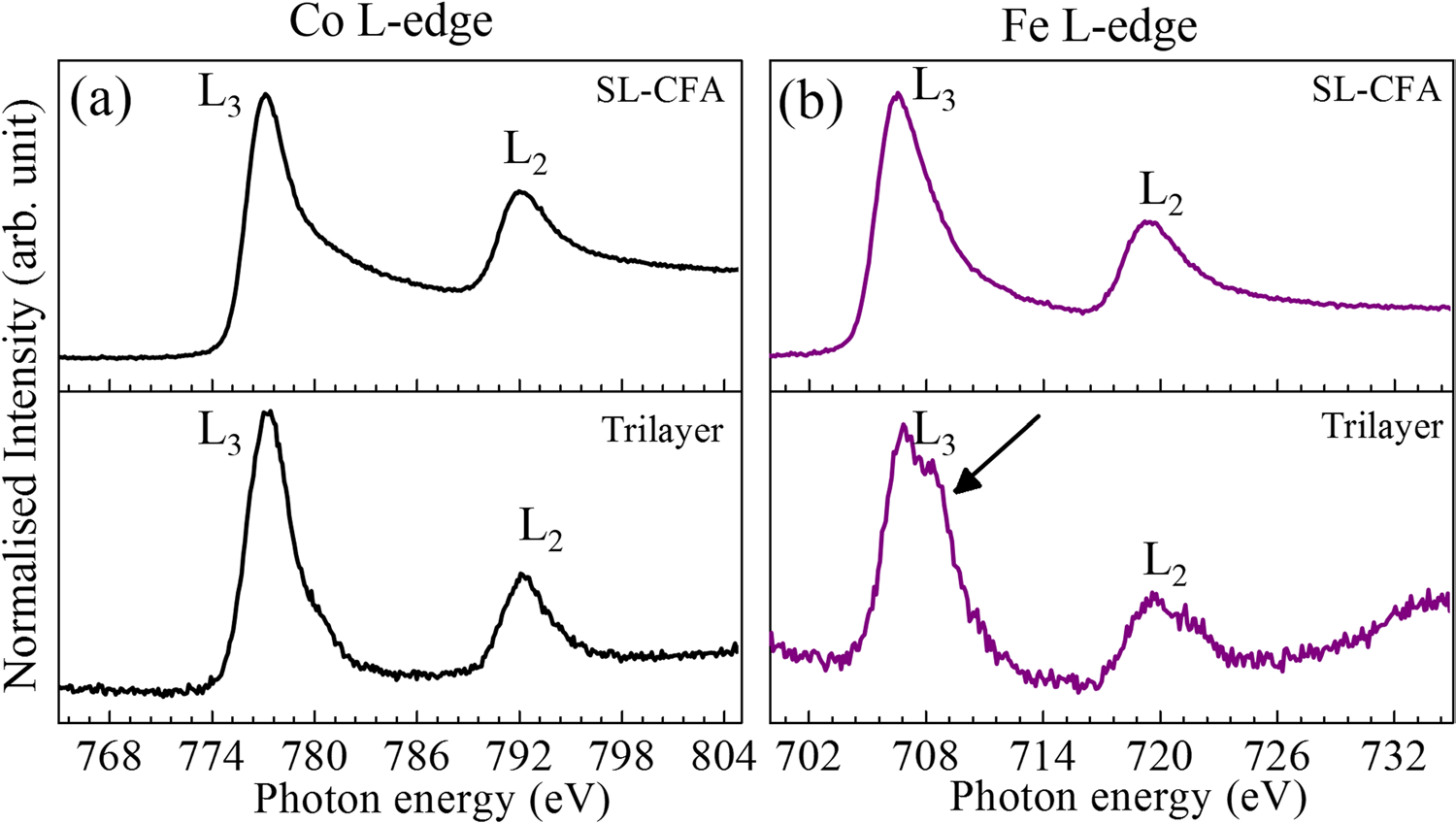
NEXAFS spectra of a single layer CFA (SL-CFA) and Ta(10)/CFA(1.8)/MgO(2) trilayer recorded at L_2,3_ absorption edges of (**a**) Co and (**b**) Fe.

The NEXAFS spectra have been recorded on Ta(10)/CFA(1.8)/MgO(2) trilayer and 50 nm thick single layer CFA (hence-after SL-CFA) thin films at room temperature. The spectra corresponding to the L_2,3_ edge of Fe and Co have been measured in total fluorescence yield (TFE) mode (see Fig. 2). The clear correspondence of each of the Co- and Fe-edge spectra between SL-CFA (top panel) and Ta(10)/CFA(1.8)/MgO(2) trilayer (bottom panel) can be clearly seen. In particular, the L_2_ and L_3_ transition peaks appear at same energy with alike broadening in the L_2,3_-edge spectra of thick (SL-CFA) and ultrathin CFA film (trilayer), which is the indication of the identical chemical environment of Co in both the films^51^. Similarly, in Fe L_2,3_-transition peaks in these spectra are identical in (both) thick and ultrathin CFA films together with the presence of shoulder (indicated by arrows in Fig. 2(b)). Consistent with the existing literature, this shoulder suggests the partial oxidation of Fe, *i*.*e*., Fe-O formation^52–54^ which is likely since MgO layer is grown over CFA layer. The clear prominence of this shoulder in the Fe L_2,3_-edges, compared to that in Co L_2,3_-edges, further suggests that at the CFA/MgO interface, the 1.8 nm CFA film terminates within the unit cell such that the terminating plane is rich in Fe.

**Figure 3:**
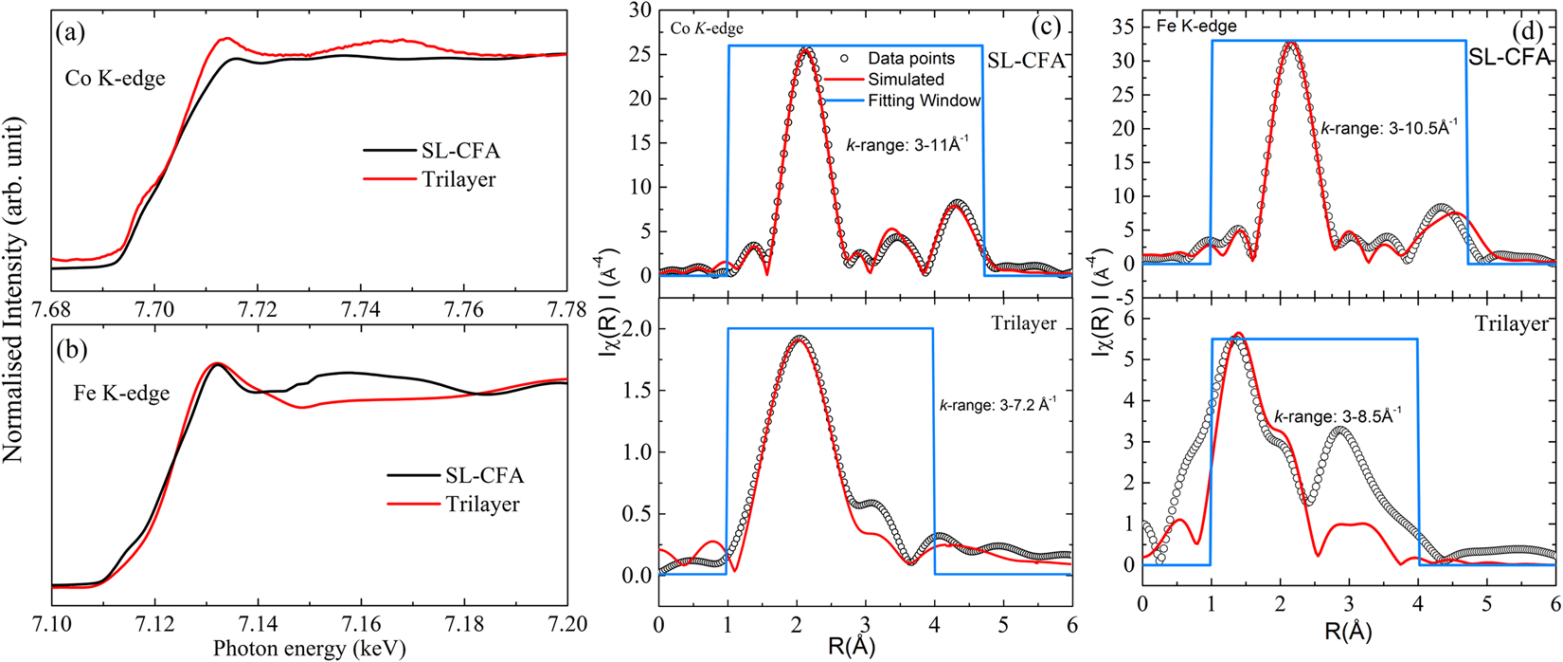
XANES spectra recorded at K-edge of (**a**) Co and (**b**) Fe on single layer CFA (SL-CFA) and Ta/CFA/MgO trilayer structure. (**c**,**d**) Present the EXAFS oscillations extracted from the X-ray absorption measurements at the Co, Fe K-edges for single and trilayer structures, respectively. The sky-blue colored box represents the region selected for the EXAFS simulation. Simulated curves are shown by red line.

Figure 3(a,b) show the X-ray absorption near edge structure (XANES) at Co and Fe K-edge on SL-CFA (50 nm) and Ta(10)/CFA(1.8)/MgO(2) trilayer, respectively. The qualitative similarity such as the maximum absorption at the same energy in the spectra (Co- as well as Fe-edge) among the trilayer and SL-CFA indicates the same local structure in them. The lower definition (low intensity) in the spectral feature of ultrathin CFA in trilayer, as compared to thick SL-CFA, is a consequence of the reduced average number of scatterers in the different coordination shells for a film at such a low thickness. Figure 3(c,d) show the χ(R), *i*.*e*., Fourier transformed (FT) absorption signal vs. radial distribution(R) spectra for SL-CFA (top panel) and trilayer (bottom panel) structures. Whereas, the two prominent peaks (near 2.2 and 4.2 Å) observed in both the Co and Fe K-edge spectra of SL-CFA represents the Co-Co [top panel, Fig. 3(c)] or Fe-Fe [top panel, Fig. 3(d)] contributions in the first nearest neighbor and Co–Fe contributions in the next nearest neighbors, respectively. The same is clearly visible in case of Co K-edge spectra of trilayer [bottom panel, Fig. 3(c)]. The shift in the locations of the two main peaks together with the qualitatively different nature of the spectra of Fe-K edge in trilayer are discussed in detail in the next para. The small intensity oscillations occurring in between these two prominent peaks arise due to the known backscattering contributions from low Z element such as Al, Mg and O. The ATOM and FEFF packages (see method for measurement and simulated fitting details) were used to generate the structural input for EXAFS data analysis^55,56^.

The simulated curves for SL-CFA (solid lines, Fig. 3(c,d)) are found to be well fitted with the experimental data which indicates that the CFA single layer thick sample is quite ordered. In Co K-edge FT spectra, the χ(R)-oscillations have a similar shape in both single and trilayer structures and the simulated parameters are also comparable which indicate the alike bonding configuration in both thick and ultrathin CFA films. In the Fe K-edge FT spectra of trilayer, the intensity variations and position of the two main peaks are different compared to that in SL-CFA. These are ascribed to the joint contributions arising as a result of the atomic disordering between Fe-Al expected to be prominent at low thickness, and the finite oxidation of Fe as is evident from the development of a clear shoulder in NEXAFS spectra (Fig. 2). The former possibility stems from the fact that Fe-Al disorder will cause local changes in the orientation of atomic planes which could be critical in films of few monolayer thickness and leads to the suppression or shifting of the main peaks^57,58^. Furthermore, since the 1.8 nm thick CFA film does not have complete unit cell (at its surface) while it terminates in contact with the MgO layer, the chemical environment is therefore quite different at its two interfaces (Mg and O at the top and Ta at the bottom). Therefore, the interference effect between the scattered waves from the two different surfaces/interfaces cannot be ignored in this trilayer structure which is known to cause multiple scattering processes, thereby contributing to the intensity and broadening of the absorption peaks^59^. The Fe-edge spectrum was therefore simulated by considering one Fe-O shell and ordered CFA structure. The sky-blue color box in the radial distribution curves represents the range (window) used for fitting simulation. The coordination numbers, bond lengths, and effect of disorder which is approximated by the Debye-Waller factor (σ^2^) (mean-square fluctuations in the path length) have been used as fitting parameters. The simulated parameters are shown in Table-II in Supplementary Information S4. At Co and Fe K-edges in CFA single layer, the coordination number and the bond length distances matched very well with the theoretical values within the uncertainty. These values are slightly different (lower) in the ultrathin film which is due to insufficient coordination shell in the ultrathin film of few monolayer thickness regime, as discussed above. Moreover, the σ^2^ is small in these films which is comparable to the reported results for ordered CFA and other Heusler compounds^60,61^.

**Figure 4:**
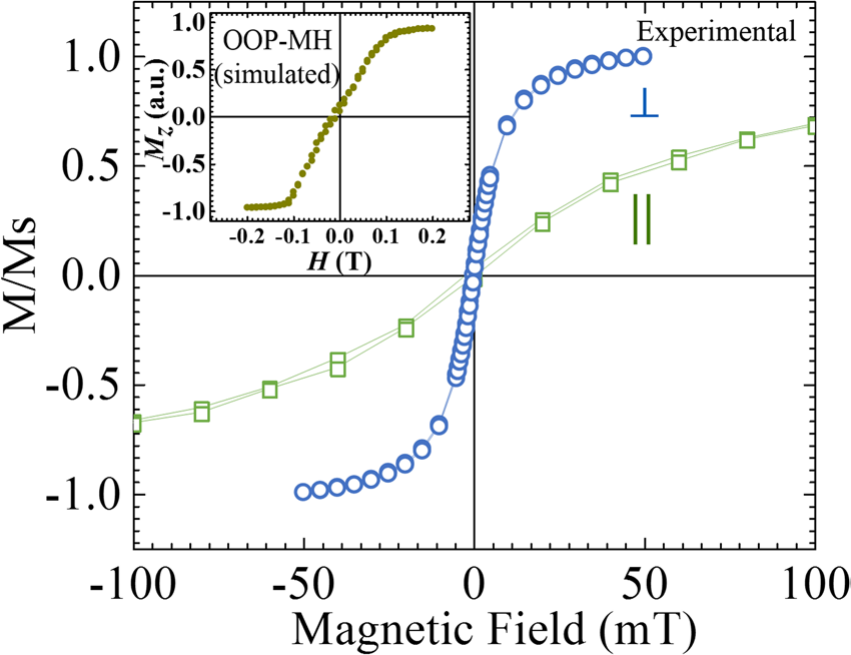
Magnetization vs. magnetic field loops recorded for the Ta(10)/CFA(1.8)/MgO(2) sample at room temperature for two magnetic field orientations, in-plane (||) and out-of-plane ($\perp$). Micromagnetic simulated out-of-plane magnetization hysteresis (OOP-MH) curve is shown in the inset.

Figure 4 shows the magnetization hysteresis (M-H) curves for the Ta/CFA/MgO trilayer heterostructure recorded both in the in-plane and the out-of-plane (OOP) magnetic field orientations. The latter M-H loop exhibits lower saturation field compared to the former suggesting that the CFA film possesses perpendicular magnetic anisotropy. The saturation magnetization is found to be 838 ± 40 kA/m which is smaller than the value of 1000 kA/m reported for the bulk samples^34^ which is due to the finite size effect in ultrathin films. The perpendicular uniaxial magnetic anisotropy energy, evaluated from the difference in the measured values of saturation fields, was found to be 2.90(±0.05) × 10^5^ J/m^3^. In the inset, the micromagnetic simulated magnetization curve, for the OOP magnetic field orientation is also shown for comparison with the experimental MH curve. Both the experimental and simulated MH curves show qualitatively similar characteristics, except for the different saturation fields which is attributed to the different demagnetization effects on account of different sample size/geometry in the experiment and simulation. The simulation details are given in Supplementary Information S1.

### Quantitative assessment of *i*-DMI at Ta/CFA/MgO Interface

We employ Brillouin light scattering (BLS) technique to determine the *i*-DMI strength in these Ta/CFA/MgO ultrathin films. The advantage of using the BLS technique is that the wave-vector of the spin wave is uniquely determined by the wavelength and the angle of incidence of the laser beam and it can simultaneously detect the propagating spin-wave excitations at +*k* and −*k* wave-vectors (Stokes and anti-Stokes processes). The signature of *i*-DMI in BLS is manifested as an asymmetry in the spin-wave dispersion relation^37,62^ for non-reciprocal propagation of Damon-Eshbach (DE) spin-waves^63^ where the spin-wave wave-vector and magnetization both lie in the sample plane and are mutually perpendicular. The *i*-DMI energy density can be determined either by modeling the full spin-wave frequency vs. wave-vector dispersion with a modified dispersion relation for DE spin waves by introducing the DMI term or from the frequency difference (*Δf*) between spin-waves with opposite (+*k* and −*k*) wave-vectors, which is given by^37^,

1 $${\rm{\Delta }}f({k}_{x})=f({k}_{x})-f(-{k}_{x})=\frac{2\gamma {k}_{x}D}{\pi {M}_{s}}.$$

**Figure 5:**
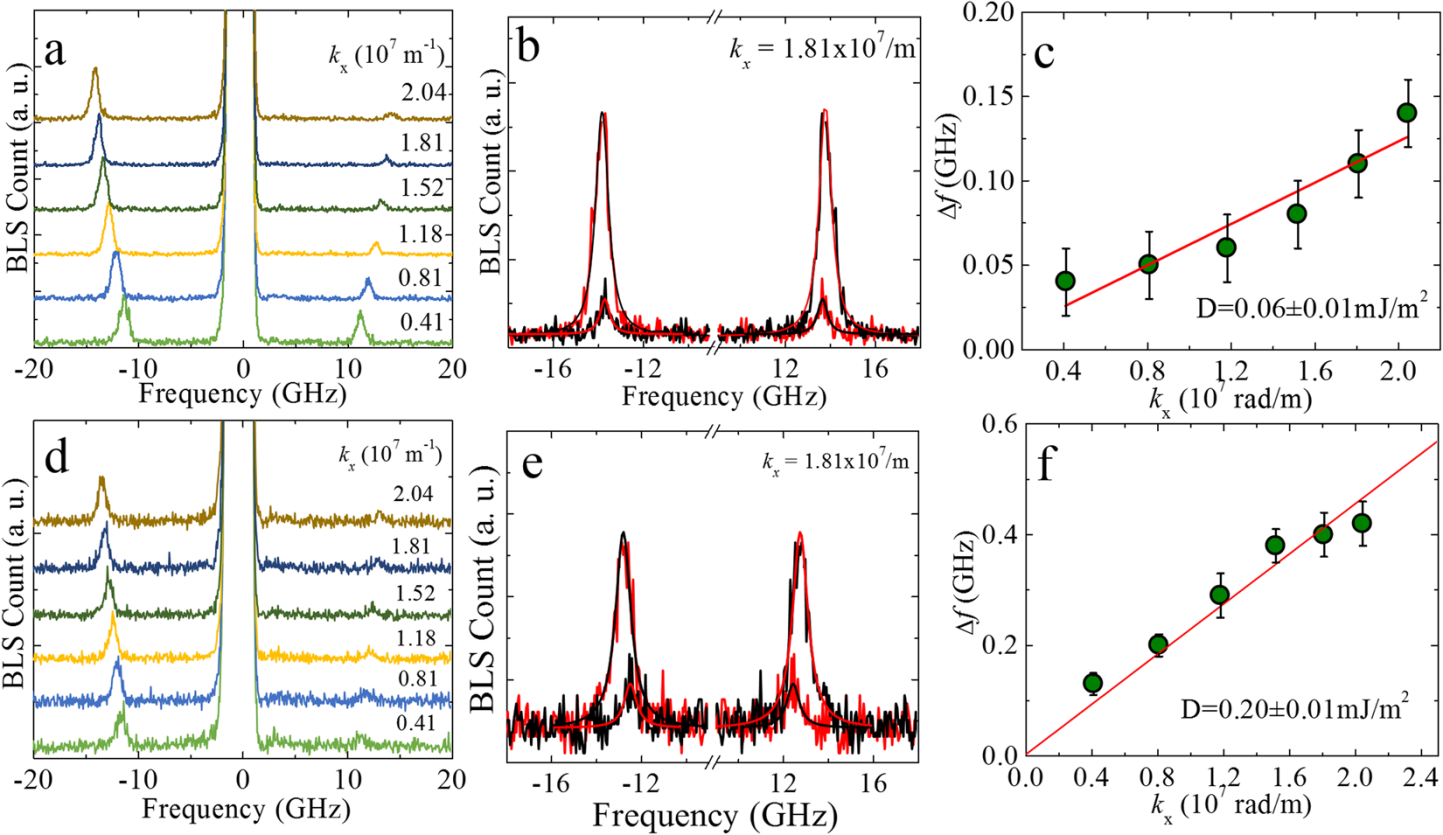
*i*-DMI in Ta/CFA/MgO ultrathin layers. (**a**,**d**) Show the BLS spectra of Ta(10)/CFA(10)/MgO(2) and Ta(10)/CFA(5)/MgO(2) thin films, respectively, recorded for various *k*_*x*_-values. The magnetic field (*H* = 0.1T) was applied in the sample plane and perpendicular to the plane of incidence of the laser beam. (**b**,**e**) Provide the Δ*f* (difference in the frequencies of the two peaks) for one of the *k*_*x*_-value such as 1.81 × 10^7^ m^−1^. (**c**,**f**) Show the Δ*f* vs. *k*_*x*_ which provide the *i*-DMI strength from its slope (given in the inset). [Lines in (**b**,**e**) are fit to the Lorentzian function and in (**c**,**f**) red lines correspond to fits using equation (1)].

Where, ${\rm{\Delta }}f$ is the frequency difference, *k*_*x*_ is the *x*-component of the wave vector, $\gamma$ is the gyromagnetic ratio, *D* is the *i*-DMI energy density and *M*_*s*_ is the saturation magnetization. In our case, we have chosen the latter method for determining *D*, as the estimation of *D* here is primarily determined by the experimentally measured quantities Δ*f*, *k*_*x*_ and *M*_*s*_(=1000 ± 50 kA/m is used for thick films^44^). Figure 5(a,d) show typical BLS spectra, recorded from Ta/CFA(*t*)/MgO thin film heterostructures with *t* = 10 and 5 nm, respectively. It is clearly observed that the positions of both the Stokes and anti-Stokes peaks in the BLS spectra move to the higher frequency values with increase in *k*_*x*_. ${\rm{\Delta }}f$ was extracted from Lorentzian fits to the BLS spectra and is plotted vs. *k*_*x*_ in Fig. 5(c,f). By fitting the experimental results by using equation (1), the strength of *D *is found to be 0.06 ± 0.01 mJ/m^2^ in Ta(10)/CFA(10)/MgO(2) and 0.20 ± 0.01 mJ/m^2^ in Ta(10)/CFA(5)/MgO(2). It can be seen that the *i*-DMI strength increases by a factor of ~4 when the thickness of CFA is reduced by half. The *i*-DMI value of 0.2 ± 0.01 mJ/m^2^ in Ta(10)/CFA(5)/MgO(2) is significantly larger as compared to the reported values for other systems with a similar thickness of the magnetic layer^37,64,65^. For the studied samples, we were unable to obtain reliable BLS signal in ultrathin CFA films (<5 nm), probably due to magnetic inhomogeneity and/or chemical/crystal ordering of the samples. However, the strength of *i-*DMI is known to be inversely proportional to the thickness of the FM layer. Hence, we anticipate a substantially higher value of *i-*DMI in lower thickness of CFA full Heusler alloy ultrathin films under investigation.

### Magnetic Force Microscopy (MFM) Imaging of Skyrmions

**Figure 6:**
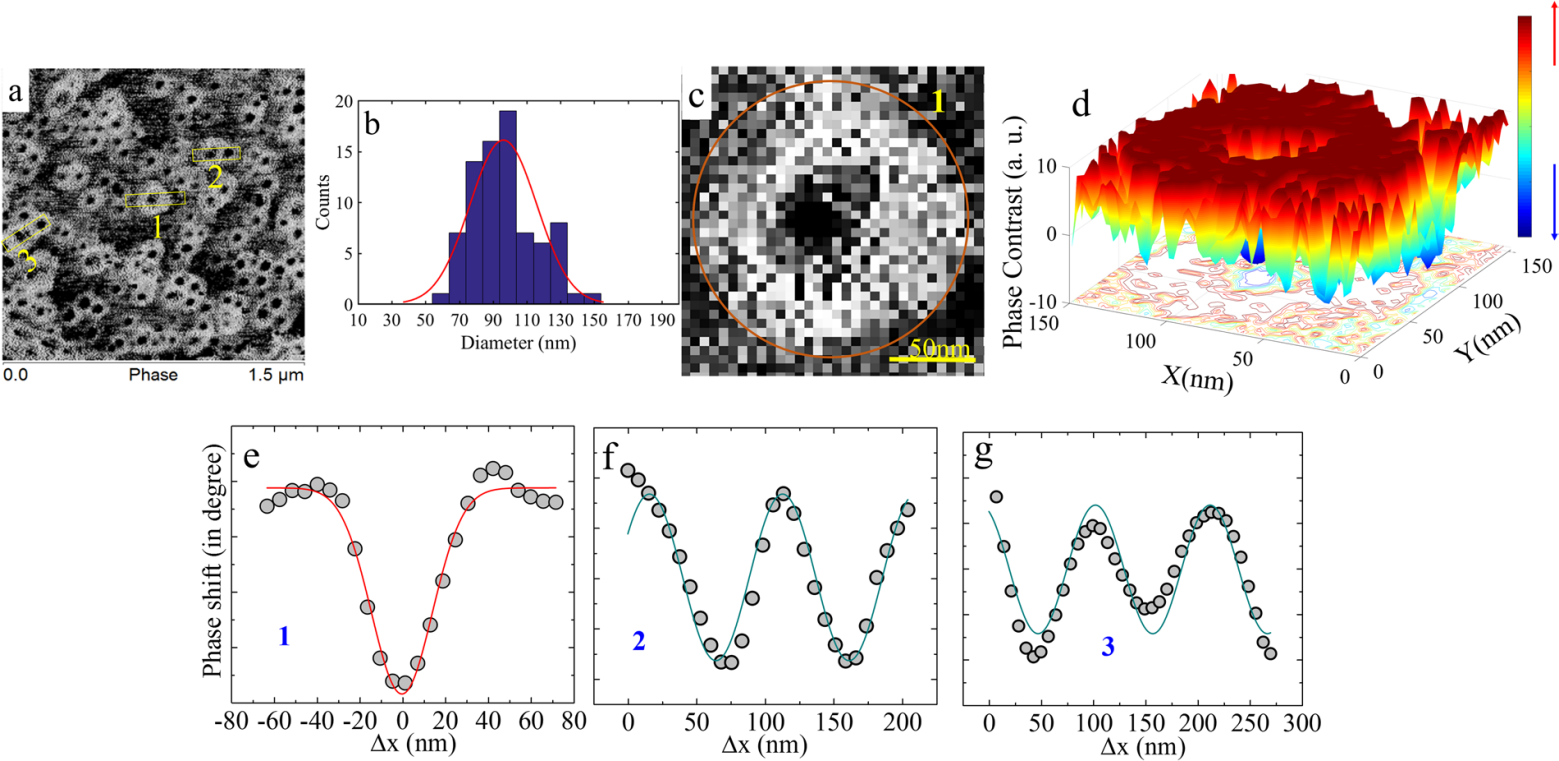
Typical magnetic force microscopic (MFM) image of the Ta(10)/CFA(1.8 nm)/MgO(2) thin film displaying room temperature magnetic skyrmions in the remnant state. (**a**) MFM image as recorded in large scan area. (**b**) The statistical distribution of the size of the magnetic skyrmions obtained by manual line scan profiling (line is the Gaussian fit to all data points). (**c**) The zoomed view of a single skyrmion labeled as 1 in (**a**). (**d**) A 3D surface plot of an experimentally observed skyrmion (different colors reveal different orientations of the magnetic flux). Lateral line scan profiling in (**e**) and fitted with simple Gaussian curve, (**f**,**g**) represent the magnetization periodicity corresponding to the label 2 and 3 marked in (**a**). (Open circles represent the experimentally obtained data points from the line scan profiling. The dark cyan line in (**f**,**g**) are the fits using a sine function for visualization of the periodicity (with period = 105 nm) of the magnetization in the case of multiple/clusters of skyrmions).

The MFM, a non-contact scanning probe, is indispensable in studies of morphology and mirostructure in terms of magnetic domains or magnetic nanostructures^66,67^. It is an important analytical tool whenever the near-surface stray-field variation of a magnetic sample is of interest. Figure 6(a) shows the MFM image (magnetic contrast) of the Ta(10)/CFA(1.8)/MgO(2) thin film recorded at room temperature in remnant state (see methods for MFM measurement details). The image displays a well-developed characteristic feature which is present throughout the whole (large) imaged area of thin film. The very uniform/confined texture with a black core is suggestive of the presence of radially chiral spin configuration in the remnant state. These structures are similar to those reported previously by other groups in different systems^25,46^. It is emphasized here that the data recorded in MFM represents the gradient of magnetic force with respect to *z*-component of the magnetic field originated from the sample. Therefore, these dense textures developed in remnant state must be chiral in nature^19,48,65,68^ which is further confirmed from the MFM simulations in the upcoming section. Further, the line (lateral) scan profiling was performed for the confirmation and characterization of the size and the periodicity of the chiral nanostructures observed in these Ta/CFA/MgO thin film. Figure 6(c) shows the zoomed area (labeled as ‘1’ in Fig. 6(a)) for a clear view of the chiral structure and Fig. 6(d) shows the corresponding 3D plot where the different color contrast corresponds to the different orientations of the *z*-component of the force gradient. Figure 6(e) shows the line scan profile performed on one such spin-texture structure within the region labeled as ‘1’ in Fig. 6(a) (circles represent data points). The magnetic contrast variation has been identified as skyrmions and to confirm our consideration we performed the identical line scan profiling on the simulated skyrmions (in the forthcoming section) and found the chirality in the observed structures; as a result, we identify/endorse them as skyrmions in these unpatterned Ta/CFA/MgO thin films at room temperature and in the remnant state. Also, the typical size of the skyrmion is found to lie in the range of ~60–150 nm. Figure 6(b) presents the skyrmion size statistics as a histogram and the average size of the magnetic skyrmions is found to be in the sub-100 nm (line represents the Gaussian fit to all data points obtained by line profiling). It may be pointed out that the observed size of skyrmions in Ta(10)/CFA(1.8)/MgO(2) is quite large as compared to the lateral resolution (~30 nm) of the employed MFM set-up which allows us to observe the skyrmions at room temperature in our specimen. Line scan profiles on multiple skyrmions in the regions 2 and 3 in Fig. 6(a) provide the magnetization periodicity and its fitting with a simple sine function yields a periodicity of 105 nm (the size of the skyrmion). The observed rotational sense of magnetization in skyrmions suggests the existence of non-zero topological (winding) number which is evaluated using the micromagnetic magnetization simulation in the following section.

**Figure 7:**
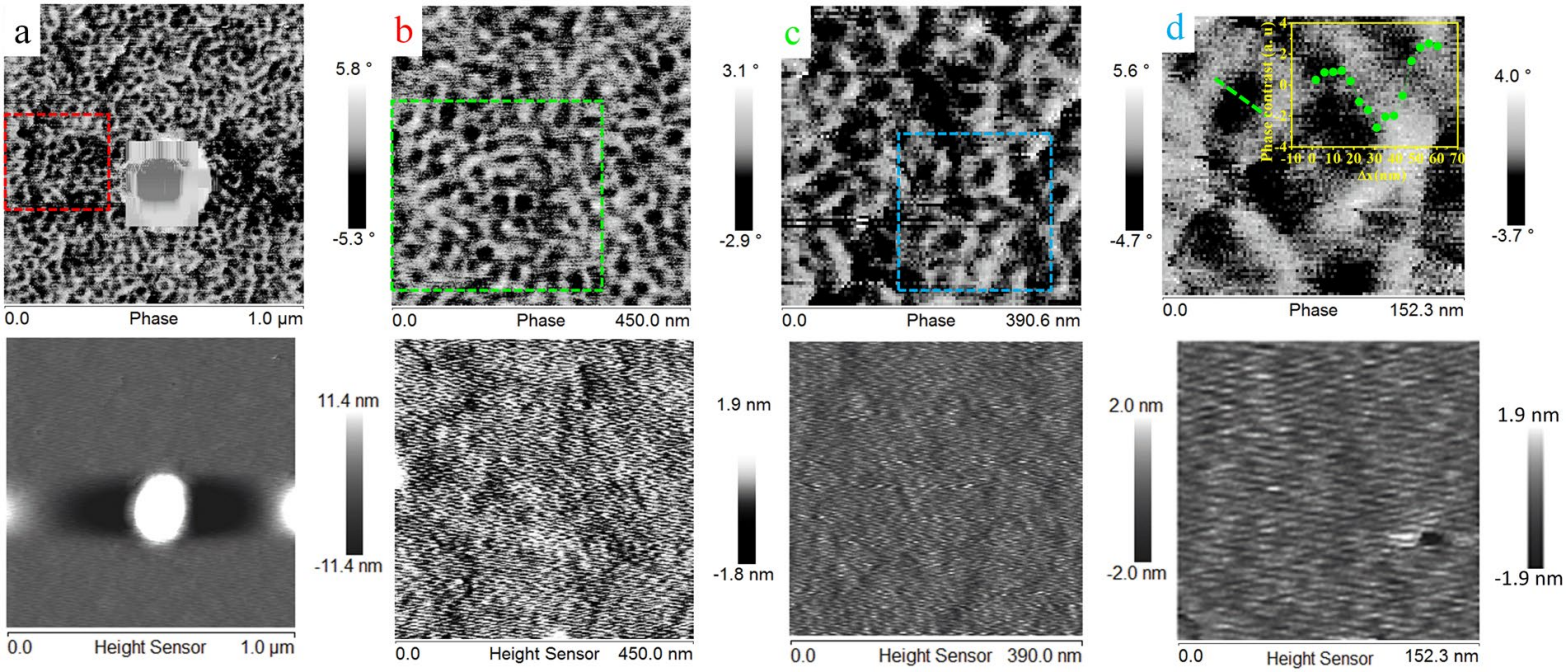
(**a**) MFM image revealing the formation of skyrmions in a Ta(10 nm)/CFA(1.0 nm)/MgO(2.0 nm) thin film heterostructure at room temperature in the remnant state achieved from +0.5 T out-of-plane magnetic field. AFM images are shown at the bottom of each MFM image. The large bright spot observed in (**a**) is due to the presence of a dust particle on the film surface. Using line scan profiling of a single skyrmion (graph shown in the inset of (d)), the size/diameter of the skyrmion is estimated to be ~50 nm by fitting using equation proposed by Romming *et al*.^29^.

Further, one of the important characteristic features of the skyrmions, the inversion of the spin-texture/contrast on reversing the direction of the magnetic field, *i*.*e*., polarity reversal^19^ in the contrast of the MFM images corresponding to the two direction of remnant magnetization has been demonstrated in the other sample, *i*.*e*., Ta(10)/CFA(1.0)/MgO(2). Figure 7 shows MFM images of the sample Ta(10)/CFA(1.0)/MgO(2) recorded in remnant state. The images reveal well-developed skyrmions at room temperature. The corresponding topography of the same scan area is shown at the bottom of the each MFM images. The signature of skyrmions is not clearly visible in Fig. 7(a) due to the large scan size. Zoomed (scanning) in portions of the image are shown in Fig. 7(b–d) revealing the formation of skyrmions texture together with a few strip-like domains which indicate that the system exhibits perpendicular magnetic anisotropy. The well-developed skyrmionic feature is clearly visible in Fig. 7(d) and the line scan profile (shown in the inset) highlights the typical size of a skyrmion.

**Figure 8:**
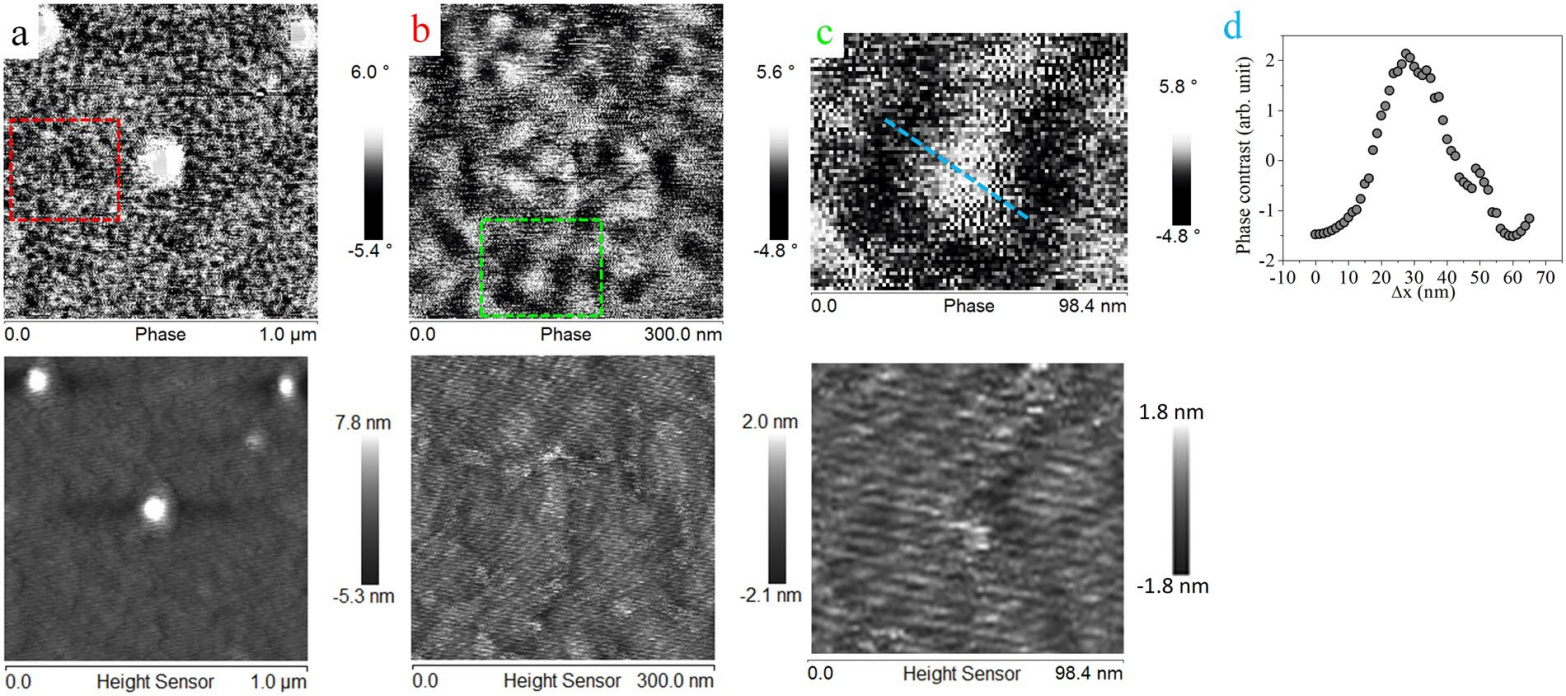
(**a**) Magnetic skyrmions in a Ta(10 nm)/CFA(1.0 nm)/MgO(2.0 nm) thin film heterostructure at room temperature in the remnant state but achieved from −0.5 T out-of-plane field, i.e., from a negatively saturated state. AFM images are shown at the bottom of each MFM image. White spots in (**a**) are due to dust particles on the film surface. (**b**,**c**) are zoomed (scans) in areas of parts marked as colored squares in (**a**,**b**), respectively. (**d**) The phase-contrast variation over a single skyrmion, i.e., across the blue line scan in (**c**). The line scan profile on the single skyrmion (blue line in (**c**)) indicates that the skyrmion has been nucleated with opposite polarity as compared to Fig. 7 where the sample was initially magnetized to saturation in a positive field.

Moreover, the skyrmions with opposite polarity are also visualized on reaching the remnant state by applying a large negative field (−0.5 T) and removed and then performed the MFM measurement. Figure 8 shows the MFM images of the Ta(10)/CFA(1.0)/MgO(2) thin film after magnetic saturation in a opposite direction as compared to the remnant state in Fig. 7. It is clearly seen that the polarity of skyrmions is reversed. Moreover, the line scan profile is shown in Fig. 8(d) indicates opposite contrast/profile (compared to the previous) in the remnant state reached after saturation in a negative field. Here, it is also realized that the domain boundaries are not distinctly resolved because of the limited MFM lateral resolution, but the skyrmionic-state is identical even in the case of a multi-skyrmions, which signifies that the structure is topologically invariant. Thus, the skyrmion formation in Ta/CFA/MgO thin films is robust against defects and shape imperfections.

**Figure 9:**
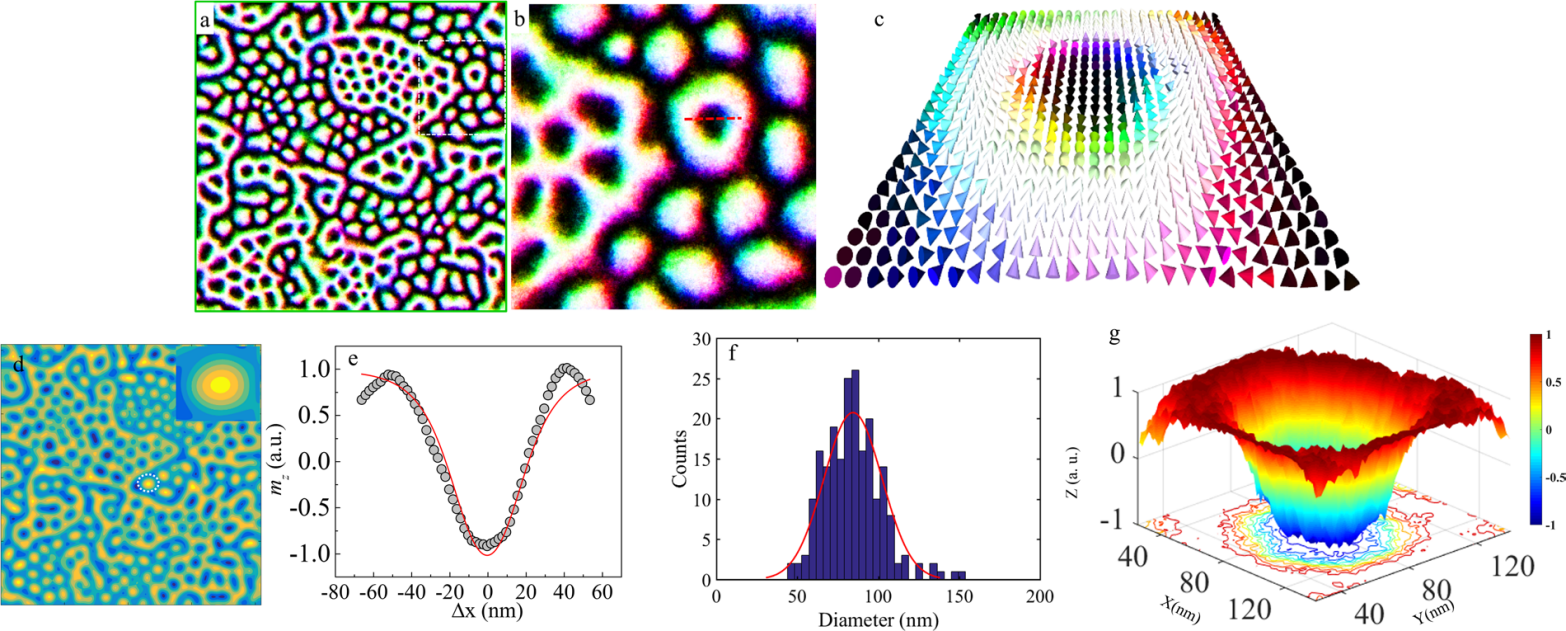
(**a**) Micromagnetically simulated stable skyrmions in an extended thin film area of 2 × 2 μm^2^, (**b**) Zoomed view of the dotted squared region from (**a**) for clarity, (**c**) A 3D-image (area 100 × 100 nm^2^) of the magnetization extracted from (**b**) shown by dotted line, (**d**) MFM simulation based on the experimentally obtained parameters (Inset: zoomed skyrmion texture) view for visualization of a single skyrmion indicating the formation of distinctly visible skyrmion. This observation of distinct spin texture qualitatively matches with the experimentally found skyrmions. (**e**) Line scan profile of a single skyrmion and the red line is fit to the equation proposed by Romming *et al*.^29^ to verify the magnetization variation within the skyrmion (open circles are the simulated points obtained using line scan profiling on the skyrmion shown by red dotted line in (b)). (**f**) The statistical distribution of the skyrmion size for the whole simulated area, and (**g**) 3D surface plot of a simulated skyrmion (extracted from (**b**) indicated by dotted line) (different colors correspond to the different polar angles of the magnetization).

To validate the experimental interpretation of room temperature skyrmions, micromagnetic simulations were carried out using the Mumax3 simulation code^69^ (see Supplementary Information S1 for simulation details). As pointed above, although the *i*-DMI could be measured only in slightly thicker films [i.e., 0.2 ± 0.01 mJ/m^2^ for CFA(5 nm)]. Assuming that the *i*-DMI strength increase inversely with the FM layer thickness^37^, for the CFA films of thickness ≤1.8 nm, the *i*-DMI strength is expected to be of ≥1 mJ/m^2^. On the other hand, in our simulations, the skyrmions were formed at the smallest DMI value of 1.2 mJ/m^2^. Thus to be consistent with experimental findings, the *i-*DMI value has been chosen to be 1.2 mJ/m^2^ for the simulations, whose results are presented in Fig. 9. We have used the saturation magnetization *M*_*s*_ = 838 kA/m as extracted from SQUID measurements. The uniaxial anisotropy strength was taken to be 3.9 × 10^5^ J/m^3^. The exchange stiffness constant (12 pJ/m) was adapted from ref.^70^. Thus, the parameters employed in the micromagnetic simulations are physically viable as they correspond to the experimentally investigated unpatterned Ta/CFA/MgO thin films. The simulations successfully realized the existence of stable single skyrmions of sub-100 nm diameter (data not shown here) as well as of the multiple skyrmions in an extended Ta(10)/CFA(1.8)/MgO(2) thin films (area ~2 × 2 μm^2^) at room temperature. Fig. 9 shows the results of these simulations in zero external magnetic field at 300 K. Figure 9(b) shows the formation of multiple skyrmions in an extended area which clearly demonstrates the features in accordance with our experimental findings (Figs. 6–8). The zoomed view of the dotted squared region of Fig. 9(a) is shown in Fig. 9(b) for clarity. A simulated 3D-image (area 100 × 100 nm^2^) of the magnetization is shown in Fig. 9(c), which clearly reveals that the observed spin texture confirms Néel type skyrmion. Further, MFM simulation was also performed in these trilayer heterostructure, which also yielded the formation of skyrmion texture (see Fig. 9(d)). Figure 9(e) shows the line scan profile of single skyrmion (shown by dotted line in Fig. 9(b)) together with a fit using the equation proposed by the Romming *et al*.^29^ to verify the magnetization variation within the skyrmion. The diameter is found to be sub ~100 nm and the statistical size distribution of the skyrmions in the image shown in Fig. 9(f) suggests a large number of skyrmions lies in the range of sub–100 nm which are comparable to the size of the skyrmions estimated experimentally from line scan profiling. The line scan profile and 3D surface plot (see Fig. 9(g)) where each color represents a different orientation of the magnetization, both reveal the chiral texture of magnetization in this system. Thus, Fig. 9(e,g) offer the wisdom that the magnetic moments point downward in the center of the skyrmion (black region) and that a re-orientation of the magnetic moments occurs as one moves towards the periphery of the skyrmion where the magnetic moments point upwards. This is consistent with the experimentally observed MFM images (Figs. 6–8) which similarly revealed the presence of annular bright regions surrounding the central dark cores. A simulated results for the sample Ta(10)/CFA(1)/MgO(2) is discussed in the Supplementary Information S1. The skyrmion number *N*_*sk*_ is also evaluated directly from the simulations for these structures. The value of *N*_*sk*_ is found to be equal to 1 which corresponds to the existence of skyrmions. Hence, the simulated magnetization and MFM structures identically match with those experimentally observed using MFM imaging performed in remnant state. Hence, it is evident that the experimentally observed spin texture in the full Heusler alloy CFA consists of clusters of skyrmions. Further, it is to be noted that skyrmion clusters have been previously demonstrated both experimentally^71–73^ as well as theoretically^73^ in patterned structures. These theoretical studies show that the single, double and even multiple skyrmions may coexist together due to skyrmion-skyrmion interactions via competing ferromagnetic exchange and DMI both of which are short-range interactions^74^.

In summary, the observation of room temperature skyrmions in ultrathin Ta/Co_2_FeAl/MgO trilayers is reported based on magnetic force microscopy imaging, and is supported by micromagnetic simulations and Brillouin light scattering measurements. The line scan profiling clearly reveals the nano-scale chiral texture. The topological state is corroborated by micromagnetic simulations by considering the experimental parameters such as interfacial Dzyaloshinskii-Moriya Interaction, exchange constant and anisotropy energy magnitudes. The study highlights the prospects of room temperature evolution of skyrmions in simple trilayer extended thin films without requiring the additional cost and time intensive film patterning constraints. The skyrmions observed in these CoFe_2_Al (1.8 nm) films were of the sub -100 nm size and even sub -50 nm in thinner Co_2_FeAl(1.0 nm) films. It is envisaged that this robust formation of skyrmions at room temperature is a vital significant experimental advancement and will pave the way towards the industrial development of the skyrmion based devices for ultra-dense memory storage and logic applications, etc.

## Methods

### Sample Preparation (Ion-assisted ion-beam sputtering)

The trilayer thin film stacks consisting of Si(100)substrate/Ta(10)/CFA(10,5,1.8,1.0)/MgO(2) (numbers in parenthesis represent film thickness in *nm*) were *in-situ* deposited in sputter-up configuration using ion-assisted ion-beam sputtering technique (after removing the native surface oxide layer of the Si substrates with HF (10:1 ratio) solution for 60 s). Prior to deposition, the vacuum chamber was evacuated by turbo molecular and cryo-pumps to a base pressure lower than ~1 × 10^−7^ Torr. A buffer layer of Ta (10) was grown at room temperature and *in situ* annealed at 773 K for 30 min in order to achieve a very flat surface. CFA layers with different thicknesses were then grown at room temperature followed by a thin layer of MgO on top of the CFA layer. A single layer CFA(50 nm) thin film was also grown for EXAFS analysis to compare with the ultrathin CFA films stacked in tri-layer heterostructure. During the growth of the Ta buffer and CFA layers, a working pressure of ~8.5 × 10^−5^ Torr was maintained during sputtering by flowing 4sccm of Ar gas. This Ar was fed through a high energy RF-ion source comprising of two-grid assembly for extraction of ~4.5″ dia Ar-ion beam. The high energy RF ion source was operated at 75 W with inner grid voltage V_**+**_  = 500 V (i.e., Ar ion-energy) and the outer grid voltage V_−_ = −270 V (for beam extraction/acceleration). The high-energy Ar ion beam so extracted was incident at an angle of 45° on 6″ dia target mounted on a water-cooled target turret. A maximum of four targets, each of 6″ dia can be mounted on the 4 sides of the turret which is rotatable *in-situ* to position any of the targets under the Ar-beam for sputtering. The target-substrate distance is ~27 cm.

For growing the MgO film, while the Mg target was sputtered using high energy RF-ion source (RF power = 100 W, V+ = 500 V and V_−_ = −270 V, Ar flow rate = 3 sccm), the growing film was simultaneously irradiated (or to say assisted) with a low energy (50 eV) oxygen ion beam (at 45° on the growing film). This oxygen beam was extracted from a low energy RF-ion source (operated at RF power = 75 W, V_**+**_ = 50 V and V_−_ = −30 V, oxygen flow rate = 12 sccm). Accordingly during the growth of MgO, whereas the total working pressure was slightly higher ~1.0 × 10^−4^ Torr, the O_2_ partial pressure was ~1.5 × 10^−5^ Torr. The deposition rates during the growth of Ta, CFA and MgO were 0.03 nm/sec, 0.03 nm/sec and 0.02 nm/sec, respectively. After the deposition of all the 3 layers, the trilayer stack was *in-situ* post-annealed at a temperature of 523 K for 1 hour in high vacuum (6 × 10^−7^ Torr).

### Characterizations

For the crystalline phase identification, a glancing incidence X-ray diffraction pattern of Ta/CFA/MgO trilayer film was recorded at a fixed glancing angle of 1° by Philip’s make X’pert(PRO) diffractometer which utilized Cu-*K*_*α*_ radiation. The deposition rates were accurately calibrated individually and then also confirmed after deposition of the stack using data from X-Ray reflectivity measurements. The thicknesses of individual layers, their density, the interface width and surface roughness were accurately estimated by simulating the XRR data.

Imaging of skyrmions in these trilayer thin films was performed in the remnant state (zero external magnetic field during imaging) by employing the *Bruker* make MFM (Model - Dimension *icon* with *ScanAsyst*). The MFM imaging was performed on the present samples to determine their remnant states after saturating (*ex-situ*) the magnetization with a perpendicular field of 0.5 T. A Co-Cr coated Si_3_Ni cantilever tip was used to map out the magnetic spin structure with a lateral resolution of 30 nm. The MFM imaging performed in this work favors the study of perpendicularly oriented magnetic domain structures. The MFM measurement consists of two steps, first tapping mode (topographical information) followed by lift mode (for magnetic domain information; positioned at the height of 70 nm from the film surface). The tip of the magnetic probe was magnetized parallel to the downward vertical direction using a permanent magnet. The resonant frequency of the cantilever (spring constant = 2.8 Nm ^−1^) was 73 kHz. Magnetization vs. magnetic field (M-H) of ultrathin films was recorded using a Quantum Design make SQUID magnetometer in both *in-plane* and *out-of-plane* configurations. The chemical valence state and the interface hybridization were examined by employing XPS. The XPS spectra on Ta(10)/CFA(1.8)/MgO(2) trilayer system were recorded using a *SPECS* make system with an Al-*K*_*α *_x-ray source (1486.6 eV) and a hemispherical energy analyzer with pass energy of 40 eV and a resolution of ~1 eV. Brillouin Light Scattering (BLS) spectra were recorded using a Sandercock-type (3 + 3) pass tandem Fabry-Pérot interferometer and a *p*-polarized (wavelength of 532 nm with 300 mW power) single longitudinal mode solid state laser. The details of the BLS set up can be found elsewhere^75^. The spectra were recorded in the conventional backscattering geometry at various wave vector orientations selected by mounting the sample on the angle-controlled sample holder providing a range of 10°−60° incident angles corresponding to wave vectors *k*_*x*_ lying in the range of 0.004–0.0204 nm^−1^. The free spectral range of 50 GHz and a 2^9^ multi-channel analyzer have been used for recording the spectra. An in-plane magnetic field of 0.1 T was applied during measurements. All measurements were performed at room temperature.

### X-ray Absorption Spectroscopy (XAS)

#### Extended X-ray absorption fine structure (EXAFS)

EXAFS spectra were at the Co and Fe *K*-edges in the step scanning mode in 1D KIST-PAL beamline of the Pohang Accelerator Laboratory, Pohang, South Korea^76,77^. To measure these spectra, higher harmonics were removed by detuning of incident beam to 60% of maximum intensity and beam energy was calibrated using reference foils (Co and Fe). Three ionization chambers filled with He and N_2_-gas were used to record the intensity of the incident and the transmitted X-rays. A fluorescence detector was also placed near to the sample holder in order to measure fluorescence current. The samples (making an angle of 45° from direction of incident X-rays) were placed between the first and second ionization chambers. The data measured in fluorescence mode was used for investigating the local atomic order.

### Simulation details for EXAFS

In order to investigate the atomic site specific information of structure and relative bond-lengths with respective to absorbing atoms, the EXAFS data was converted into radial distribution function χ(R) (Fourier transformed into R-space) after background subtraction as a function of effective distance R (in Å). Here, R includes not only the interatomic distances but also the scattering phase shifts also. IFEFFIT/ARTEMIS^78^ were used to simulate/process the recorded data with the graphical interface Athena^55^. The theoretical structure for fully ordered Co_2_FeAl full Heusler compound was generated using the ATOM and FEFF^55,56^. The errors in the fit parameters σ^2^ were obtained from 90% of happiness factor.

#### Near edge X-ray absorption fine structure (NEXAFS)

The NEXAFS spectra were recorded at soft X-ray beam line 10D KIST beam line^79,80^. The system was evacuated to ~1.5 × 10^−8^ Torr. The XAS spectra from the single layer CFA(50 nm) and trilayer Ta(10 nm)/CFA(1.8 nm)/MgO(2 nm) ultrathin films were recorded in total fluorescence yield (TFY) mode. Fixed voltage (1.75 kV) was applied to micro-channel plate for recording the spectra in TFY mode and the fluorescence detector (40 mm diameter) was placed at an angle of 45° from direction of beam axes. The incident photon flux was measured by inserting the gold (Au) mesh in the path of X-ray beam.

## Electronic supplementary material

Supplementary file
