# Supplementary material for: Observation of Skyrmions at Room Temperature in Co2FeAl Heusler Alloy Ultrathin Film Heterostructures
Source: Sci Rep. 2019 Jan 31;9:1085. doi: 10.1038/s41598-018-35832-3 (PMC6355792; doi:10.1038/s41598-018-35832-3)
Supplement: Supplementary file 1 — Supplementary file [file 41598_2018_35832_MOESM1_ESM.pdf]

Supplementary Information

Observation of Skyrmions at Room Temperature in Co_2FeAl Heusler Alloy Ultrathin Film Heterostructures

Sajid Husain,[1] Naveen Sisodia,[1] Avinash Kumar Chaurasiya,[2] Ankit Kumar,[3] Jitendra Pal Singh,[5] Brajesh S. Yadav,[4] Serkan Akansel,[3] Keun Hwa Chae,[5] Anjan Barman,[2] P. K. Muduli,[1] Peter Svedlindh,[3] and Sujeet Chaudhary[1,*]

[1]*Thin Film Laboratory, Department of Physics, Indian Institute of Technology Delhi, New Delhi 110016, India*

[2]*Department of Condensed Matter Physics and Material Sciences*

S. N. Bose National Centre for Basic Sciences

Block – JD, Sector – III, Salt Lake, Kolkata – 700106, India

[3]*Department of Engineering Sciences, Uppsala University, SE-75121, Uppsala, Sweden*

[4]*Solid State Physics Laboratory, Lucknow Road, Timarpur, Delhi 110054, India*

[5]*Advanced Analysis Center, Korea Institute of Science and Technology, Seoul 02792, Republic of Korea*

S1 Micromagnetic Simulations:

The micromagnetic simulations were performed using the mumax 3.8 simulation tool[1] integrated with the Landau-Lifshitz-Gilbert (LLG)

$$\frac{\partial M_s}{\partial t} = -\gamma M_s \times H_{eff} + \alpha M_s \times \frac{\partial M_s}{\partial t},$$

where γ is a gyromagnetic ratio, α is the Gilbert damping constant, M_s is the saturation magnetization and H_{eff} is the local effective field consisting of as the following contributions,

$$
\begin{aligned}
H_{eff} &= H_{exc} + H_{ani} + H_{dmg} + H_{ext} + H_{dmi} \\
&= \frac{2A_{ex}}{\mu_0 M_s}\left[\left(\partial m_x / \partial x\right)^2 + \left(\partial m_y / \partial y\right)^2 + \left(\partial m_z / \partial z\right)^2\right] + \frac{2K_u}{\mu_0 M_s} - \mu_0^{-1} M_s (m_x N_x + m_y N_y + m_z N_z) \\
&\quad + H_{ext} + \frac{2D}{\mu_0 M_s}\left(\frac{\partial m_z}{\partial x} + \frac{\partial m_z}{\partial y} - \left(\frac{\partial m_x}{\partial x} + \frac{\partial m_y}{\partial x}\right)\right).
\end{aligned}
$$

Where, H_{iDM} is the field due to DM interaction which is primarily responsible for curling of the magnetization and for skyrmion formation. In addition to above terms, a fluctuating thermal field H_{TH} is included which controls the temperature effect in the micromagnetic system. It is modeled by a Langevin random field $H_L(H_{L_x}, H_{L_y}, H_{L_z})$ where each component follows a zero-mean Gaussian random process whose standard deviation is a function of temperature which is defined by $\delta = \sqrt{2\alpha k_B T / \gamma\mu_0 M_s v_F \Delta t}$ (k_B is a Boltzmann constant, v_F is the micromagnetic cell volume, T is the temperature, and Δt is the integration time-step. The boundary conditions were taken care of by the simulation program as given in ref.[1].

For simulation of the extended thin film, a square-shaped geometry with dimensions 2048 nm×2048 nm was considered. The finite difference computational cell/grid size was kept constant at 2 nm as discrete rectangular grids with constant thickness of 1−4 nm considered to be effective thickness, i.e., the sum of the thicknesses of FM and the adjoining layer and is assumed to be optimum for the relevant micromagnetic length scales[2]. The damping constant was kept fixed to 0.01. The skyrmionic state was observed in particular combinations of parameters which were determined with the help of our experimental results.

The simulations were performed starting with an initial random magnetization and then relaxed the system (both in zero magnetic field), which eventually resulted in a magnetization configuration corresponding to the skyrmions state. However, when the relaxation to eventual

magnetization configuration was simulated using an initial state corresponding to the different out-of-plane magnetic field (i.e., non-remnant state), it was observed that the skyrmions start disappearing on above 100mT. The simulations show that for fields above 500mT, the final state corresponds to ferromagnetic continuum state.

S1(I): Skyrmions in ultrathin films

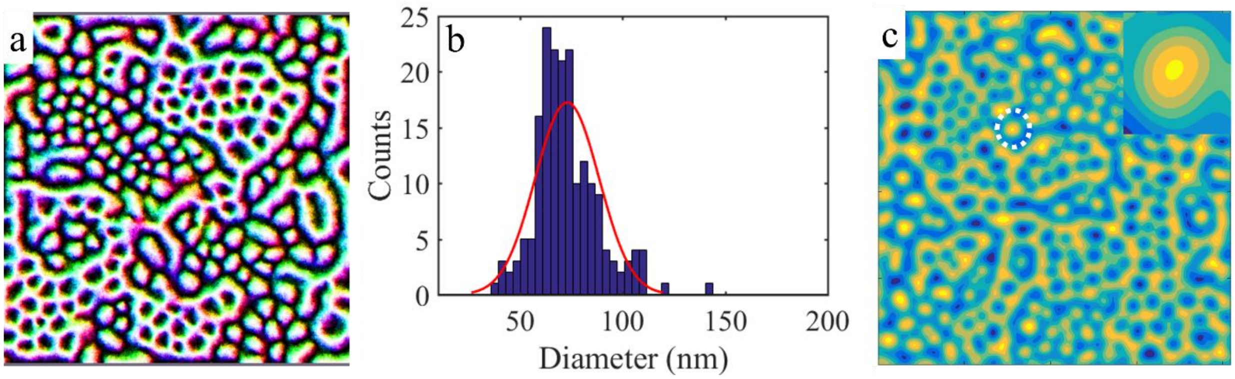

Figure S1**:** Micromagnetic simulated stable skyrmions in Ta(10)/CFA(1)/MgO(2) thin film stack of area 2×2 μm^2, keeping K_u and *i*-DMI same as in the other simulations. (b) The statistical distribution of the skyrmions diameter (red line is Gaussian fit to the data). (c) Simulated MFM image for Ta(10)/CFA(1)/MgO(2) stack (Inset: zoomed view of skyrmion shown by dotted circle).

S1(II) Comparison of skyrmion diameter (experimental and simulated)

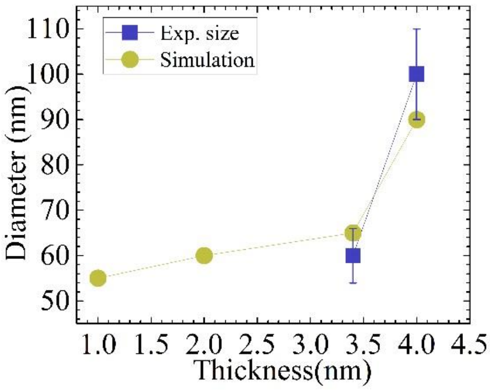

Figure S2. Experimental and simulated skyrmion diameters in Ta/CFA/MgO thin films vs. effective FM layer thickness. Error bars in the experimental data are due to the standard deviation in at least 10 individual measurements using line-scan profile. The simulation was also done for lower thicknesses i.e., 1.0 and 2.0 nm and diameter plotted herewith (simulated images not shown here). The variation of the diameter is indicating that the tuning of the FM layer thickness can be useful for obtaining skyrmions within sub-50 nm diameter.

S2 X-ray reflectivity for thickness and interface width measurement

Figure S3 shows the simulated (solid line) and the experimental (data symbol) specular XRR spectra of the CFA thin films of two thicknesses. The simulated parameters are shown in Table. I. The XRR spectra illustrate the distinct presence of *Kiessig* fringes over the entire range of incident angle clearly indicating the presence of sharp and high quality interfaces.

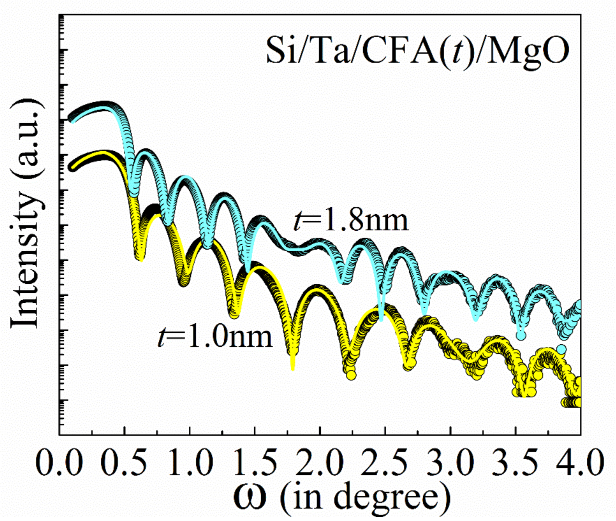

Figure S3: Specular X-ray reflectivity spectra of Si/Ta(10)/CFA(*t*)/MgO(2) thin films. The symbols are experimental data and the solid lines represent the simulated spectra obtained by a segmented fitting model. The simulated parameters are tabulated in Table.I.

Table. I: Simulated parameters of Si/Ta(10)/CFA(*t*)/MgO(2) thin film samples.

	***t*=1.8nm**			***t*=1.0nm**		
**Layer**	ρ(gm/cc)±0.06	*t*(nm)±0.03	σ(nm) ±0.03	ρ(gm/cc)±0.06	*t*(nm)±0.02	σ (nm) ±0.03
SiO_2	02.00	02.55	0.21	01.90	02.47	0.34
Ta	12.67	10.95	0.50	14.44	10.31	0.35
CFA	07.50	01.68	0.28	07.06	01.00	0.30
MgO	02.50	02.28	0.48	02.64	02.78	0.16
MgO_x	02.00	01.42	0.33	01.65	01.12	0.23

S3 Interfacial Hybridization at CFA/MgO interface (Origin of SOC at interface for PMA)

It is well known that the perpendicular magnetic anisotropy (PMA) in FM/transition metals (TM) capped with MgO is attributed to the hybridization of O 2*p* orbitals with either Co 3*d* or Fe 3*d* orbitals at the TM/MgO interface[3,4]. Here, we present the evidence of PMA in CFA/MgO whose origin is attributed to the interfacial interaction between the FM and the adjacent oxide layers. The

interaction between these two layers provides the hybridization which leads to enhanced spin-orbit coupling (SOC) at the CFA/MgO interface. To ascertain such hybridization in the present case, XPS spectra were recorded on the CFA(1.8)/MgO(2) thin film stack. The XPS spectra were corrected using the C 1*s* peak and deconvoluted using the XPS-peak 4.1 software. Fig. S4(a) shows the Mg 2*p* spectrum where the peak at 50.6 eV designates the Mg^{2+} state corresponding to MgO with no signal corresponding to Mg-metal. This ensured the formation of stoichiometric MgO during the deposition of the MgO layer. In Fig. S4(b) the asymmetric O 1*s* peak is deconvoluted into two component peaks centered at 531.7 eV(OI) and 529.6 eV(OII), respectively coming from the interstitial O^- state corresponding to the formation of MgO and CoO at the interface[5,6,7]. The Co 2*p* electronic spectrum shown in Fig. S4(c) is also shown deconvoluted into two peaks, namely the Co 2*p* as metal and Co 2*p* as CoO at 780.04 eV and 778.35 eV, respectively. Thus, the presence of interfacial hybridization (which is essential for large SOC) of O 2*p* orbitals with Co 3*d* orbitals at the CFA/MgO interface is clearly evidenced in these samples.

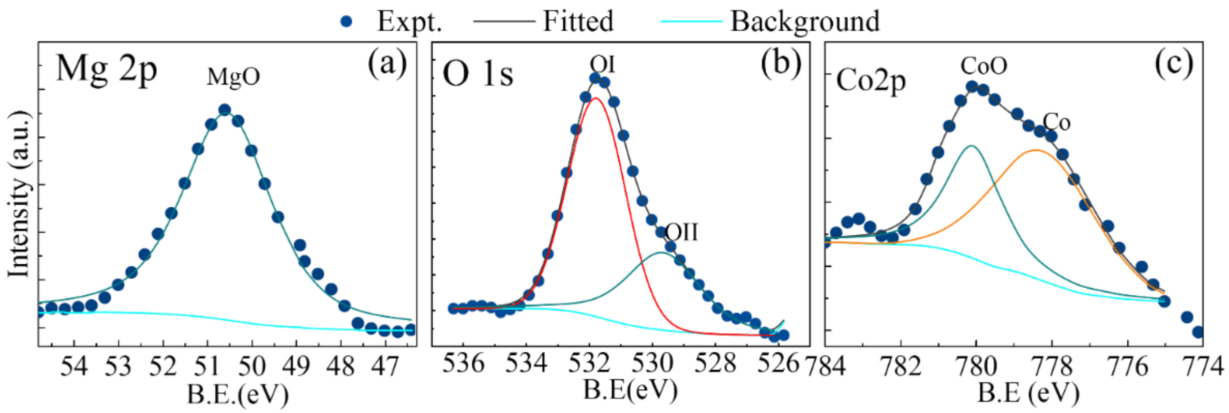

Figure S4: XPS spectra recorded on the Ta(10)/CFA(1.8)/MgO(2) thin film: (a) The MgO 2p peak shows the complete oxidation of Mg because of the absence of the Mg metal peak. (b) The interstitial O^- ions peak appear due to interstitial oxygen in MgO (OI) and the shoulder indicates the metal-oxide formation (OII). (c) The clear observation of the

CoO $2p_{3/2}$ peak establishes the presence of the interfacial hybridization at the CFA-MgO interface.

S4 Extended X-ray absorption fine structure (EXAFS) at Co and Fe K-edge

Table II: The simulated parameters such as bond length (R), coordination number N, and disorder factor (σ^2) obtained by EXAFS fitting for Co_2FeAl at (A) Co K-edge, and (B) Fe K-edge for both 50 nm thick single layer CFA (SL-CFA) and Ta(10)/CFA(1.8)/MgO(2) trilayer.

A. Co *K*-edge

	CFA single layer					Trilayer		
Shell	N	N*	R (Å)	R*(Å)	σ^2 (Å^2)	N	R (Å)	σ^2 (Å^2)
Co-Al	4.3±0.3	4	2.429±0.005	2.448	0.003±0.002	2.8±0.2	2.32±0.02	0.015
Co-Fe	4.3±0.3	4	2.457±0.008	2.448	0.002±0.001	2.8±0.2	2.32±0.02	0.015
Co-Co	6.4±0.3	6	2.883±0.050	2.827	0.011±0.004	4.2±0.2	2.69±0.02	0.015

B. Fe *K*-edge

	CFA single layer					Trilayer		
Shell	N	N*	R (Å)	R*(Å)	σ^2 (Å^2)	N	R (Å)	σ^2 (Å^2)
Fe-Co	8.4±0.2	8	2.49±0.01	2.448	0.004±0.001	4.5±0.2	2.39±0.03	0.022
Fe-Al	6.3±0.2	6	2.45±0.06	2.827	0.039±0.013	3.3±0.2	2.77±0.03	0.022
Fe-Fe	12.7±0.2	12	4.35±0.06	3.998	0.017	6.7±0.2	3.94±0.03	0.022
Fe-O						4.8±0.8	2.03±0.01	0.001

*Theoretical values (of N and R) for the ordered CFA Full Heusler alloy

Supplementary References:

1. Vansteenkiste, A. *et al.* The design and verification of MuMax3. *AIP Adv.* **4,** 107133 (2014).

2. Woo, S. *et al.* Observation of room-temperature magnetic skyrmions and their current-driven dynamics in ultrathin metallic ferromagnets. *Nat. Mater.* **15,** 501–506 (2016).

3. Yang, H. X. *et al.* First-principles investigation of the very large perpendicular magnetic anisotropy at Fe|MgO and Co|MgO interfaces. *Phys. Rev. B* **84,** 054401 (2011).

4. Baumann, S. *et al.* Origin of Perpendicular Magnetic Anisotropy and Large Orbital Moment in Fe Atoms on MgO. *Phys. Rev. Lett.* **115,** 237202 (2015).

5. Singh, B. B., Agrawal, V., Joshi, A. G. & Chaudhary, S. X-ray photoelectron spectroscopy and conducting atomic force microscopy investigations on dual ion beam sputtered MgO ultrathin films. *Thin Solid Films* **520,** 6734–6739 (2012).

6. Zhang, J. Y. *et al.* Effect of MgO/Co interface and Co/MgO interface on the spin dependent transport in perpendicular Co/Pt multilayers. *J. Appl. Phys.* **116,** 163905 (2014).

7. Lopez-Santiago, A. *et al.* Cobalt ferrite nanoparticles polymer composites based all-optical magnetometer. *Opt. Mater. Express* **2,** 978 (2012).
